# Understanding safe sport as a public health practice: a strong structuration theory analysis of Swedish elite athletics sport personnel

**DOI:** 10.1186/s12889-026-29709-8

**Published:** 2026-09-30

**Authors:** Carolina Lundqvist, Moa Jederström, Laura Korhonen

**Affiliations:** 1 Department of Behavioural Sciences and Learning, Linköping University, Linköping, SE-581 83 Sweden; 2 Athletics Research Center, Department of Health, Medicine and Caring Sciences, Linköping University, Linköping, Sweden; 3 Clinical Department of Child and Adolescent Psychiatry in Linköping, Region Östergötland, Linköping, Sweden; 4 Barnafrid and Department of Biomedical and Clinical Sciences, Linköping University, Linköping, Sweden

**Keywords:** Athlete welfare, Elite Sport, Mental health, Violence prevention, Olympic, Safeguarding, Sport governance, Sport integrity, Wellbeing

## Abstract

**Background:**

Exposure to harmful environments and practices is reported across a wide range of sports, including athletics, with negative implications for participants’ health and welfare across age groups, genders, and competitive levels. Sport environments are shaped by powerful cultural norms and organisational practices, which influence how sport personnel responsible for shaping elite athletes’ sporting environments understand, prioritise and enact safety in practice. This study explores how sport personnel in Swedish elite athletics interpret and enact safe sport, and how these enactments are shaped, enabled, and constrained by structural conditions.

**Methods:**

Sport personnel (*n* = 17; 8 women, 9 men; aged 35–66) with responsibility for shaping elite athletes’ sporting environments, including teachers and coaches at national elite sport high schools, managers of high-performance centres and national team head coaches, within Swedish Athletics were purposively sampled across elite organisational levels (i.e., national elite sport high schools, high-performance centres, national teams). Data were generated through semi-structured interviews. Analysis was conducted using reflexive thematic analysis within a social constructivist-informed qualitative framework. Stones’ Quadripartite Nature of Structuration model, an extension of Strong Structuration Theory, informed the organisation and interpretation of themes and subthemes.

**Results:**

Three themes describe how safe sport was understood, enacted, constrained, and transformed within Swedish elite athletics. Theme 1 addresses how sport personnel understood safe sport through the interplay between internal and external structures, including holistic understandings, temporal shifts, and resource conditions. Theme 2 reflects active agency through everyday practices, including collaborative agency and micro-level interventions. Theme 3 highlights structural constraints and opportunities for safe sport transformation, including solitary coaching practices, power asymmetries, institutional silence, performance norms that normalise harmful behaviours, fragmented accountability structures alongside ongoing efforts toward institutionalisation.

**Conclusions:**

Sport personnel described safe sport as extending beyond safeguarding and the prevention of harm to include broader organisational and sociocultural conditions that support athlete welfare. The findings position safe sport as a settings-based public health practice contingent on organisational conditions that strengthen sport personnel’s agency and enable preventive action. Transformative change towards safe sport appears to require institutionalised prevention capacity, including clear accountability, and resources that extend beyond individual sport personnel’s awareness and commitment.

**Supplementary Information:**

The online version contains supplementary material available at https://doi.org/10.1186/s12889-026-29709-8.

## Background

Athletics (i.e. track and field), governed internationally by World Athletics and a cornerstone sport of the Olympic Games, is a cluster of disciplines (e.g., sprinting, middle- and long-distance running, hurdles, jumps, throws, and combined events) and comprises more than 200 national federations [1]. Athletics is generally not regarded as a high-risk sporting context for maltreatment, but studies involving international cohorts have still documented exposure to sexual, physical, and psychological abuse among athletes [2, 3]. Also more broadly, the occurrence of harmful sport environments has been reported across a wide range of sports, with negative implications for participants’ physical and mental health, wellbeing, continued participation and athletic performance across ages, genders, and competitive levels [2–6]. Efforts to prevent such harms and promote athlete welfare are increasingly framed within the concept of “safe sport” [7]. International and national governing bodies, including World Athletics [8], have therefore increasingly prioritised safe sport as a strategic priority, which reflects increasing commitment to preventing violence and misconduct in sport while safeguarding athlete welfare and the integrity of sport.

### Challenges in safe sport research and practice

Research about the ethical responsibility to protect sport participants from abuse, exploitation and avoidable harm predates the contemporary terminology of safe sport. Historically, these responsibilities have commonly been addressed through safeguarding, child protection, professional conduct and organisational duty of care [9, 10]. Despite increasing global awareness of safe sport, the research field remains conceptually and methodologically fragmented. Across research and policy contexts, the terms safe sport, safeguarding and sport integrity are frequently used interchangeably or in combination [11]. These concepts share a focus on both preventing harm and promoting positive experiences in sport. Safe sport is generally understood as an overarching framework that encompasses both safeguarding and sport integrity perspectives. It refers to the creation of sport environments in which participants can thrive through the promotion of welfare, inclusion, fairness, enjoyment, personal development and human rights [9, 12]. A central challenge with the concept of safe sport environments is its inherently broad and evolving scope that risks encompassing most challenges in sport and leads to varying interpretations across cultures, contexts, and populations [12]. Safeguarding relates primarily to protective measures to prevent and respond to harm such as interpersonal violence and exploitation, while also promoting athlete welfare [9]. Safe sport and safeguarding have also been linked to sport integrity, understood as ethical governance and the principles of fairness, accountability, transparency and trustworthiness within sport organisations, which is considered important for sports’ legitimacy, reputation and growth [11, 12]. In the present study, safeguarding refers to policies, procedures, and practices intended to prevent and respond to harm, whereas safe sport is understood more broadly as encompassing the organisational and sociocultural conditions that support safe, healthy, and inclusive sporting environments. Safeguarding is therefore treated as one component of safe sport rather than as an equivalent concept.

Without clear boundaries and agreed definitions around safe sport, it becomes challenging to establish shared priorities, responsibilities and standards across sport systems. Recent concerns have also been raised regarding the absence of coherent, evidence-informed frameworks, and robust oversight mechanisms [12–14]. Existing research is largely descriptive with substantial heterogeneity, which, coupled with tendencies toward conceptual creep across integrity and maltreatment domains, may dilute conceptual clarity and analytical focus [6, 7, 12, 13]. This is particularly important because sport organisations have repeatedly been described as resistant to systemic change, often responding to crises with short-term policy initiatives that weaken over time as external scrutiny diminishes [7]. Cultural norms, values and organisational practices, often described as “sport culture”, contribute to the conditions under which harmful practices, including psychological abuse and other forms of interpersonal violence, can be normalised, tolerated and even justified [5–7]. In this study, sport culture refers to broader shared norms, values and expectations within athletics, whereas organisational practices refer to the everyday routines, actions, and governance arrangements through which these norms are enacted. These cultural norms and values may be embedded in decision-making processes, power-hierarchies, and sport organisational structures and systems of accountability, conditioning and shaping conformity to dominant values within the sport system, even when practices are identified as unsafe or unacceptable by external stakeholders or society more broadly [15–17]. Consequently, sport personnel working within institutionalised norms may be influenced in how they communicate about and respond to concerns, allocate responsibility, and prioritise athlete welfare [6, 16, 17]. Difficulties in implementing and sustaining safeguarding measures may therefore not only reflect individual resistance but also broader sport (sub)cultural and organisational norms, beliefs or values that shape expectations and behaviours. Over time, perceived institutional neglect and weak accountability can erode organisational trust [18, 19], contributing to increasing scepticism towards the value of safeguarding initiatives and questioning participants’ disengagement from broader societal standards [12, 20]. Accordingly, the present study focuses on how safe sport is interpreted and enacted as a normative, organisational, and sociocultural practice.

### Safe sport as a public health challenge

Organised sport has long been recognised as an important setting for promoting physical and mental health and social inclusion [21, 22], which aligns with the World Health Organization’s [23] definition of health setting as: *“The place or social context where people engage in daily activities*,* in which environmental*,* organizational and personal factors interact to affect health and well-being”* (p. 30). Beyond participation itself, elite sport has also been used as a platform for promoting healthier lifestyles within the wider population, although the general population health effects of elite sport are debated [24]. Given that sport engages a substantial proportion of the population across the lifespan, it represents an important public health setting [25] in which participants should be afforded opportunities for development, wellbeing and performance while being safeguarded from physical, psychological and social harm. The outcomes of sport participation, whether beneficial or harmful, depend however on the conditions under which participation takes place and how these conditions are experienced.

Historically, organised sport has been characterised by a strong tradition of self-governance, with sport governing bodies seeking to preserve their autonomy from external actors and maintain authority over their own rules, policies, and organisational structures [26]. This autonomy has been justified by the assumption that sport is inherently beneficial and socially desirable [27]. Similarly, public investments in elite sport programmes and major sporting events have often been justified by claims that elite sport generates broader societal benefits, including increased physical activity and sport participation among the general population through inspiration and improved sport infrastructure [28]. However, a growing body of research challenges these assumptions [24]. Current evidence provides little support for the notion that a nation’s elite sport success or hosting of elite sport events leads to meaningful increases in population-level physical activity or sport participation, unless accompanied by targeted public health initiatives [24, 28–32]. Consequently, claims regarding the public health value of elite sport warrant greater critical scrutiny. In sport, athletes are repeatedly exposed to norms, relationships, organisational routines, material resources and governance arrangements that may either protect their health and welfare or reproduce risks of violence, psychological harm, injury, reduced help-seeking and institutional silence [6, 33–35]. Safe sport therefore becomes a public health concern when harm is understood not only as individual violations, but as patterned and preventable outcomes shaped by the sport settings [36]. Violence and inadequate access to support can be understood as determinants of athlete health [9, 36]. A public health approach consequently directs attention upstream to the organisational capacity to prevent, identify and respond to harm [36]. This settings-based perspective is particularly relevant in youth and elite sport settings, where athletes may spend extensive time in training, competition, educational and residential environments. In such contexts, health and safety are shaped not only by individual behaviours, but also by structures that organise authority, dependency, accountability, selection, access to support and possibilities of disclosure [6, 33–35]. Safeguarding policies are therefore necessary but may be insufficient if sport personnel lack the capacity to enact them or they remain symbolic [11]. Understanding safe sport as a public health practice requires examination of how prevention is interpreted and enacted within these everyday organisational conditions.

### Study rationale and aim

While much of the existing literature has focused on safeguarding, maltreatment, and governance failures [2–4, 7, 9, 17, 20], less attention has been directed towards how sport personnel responsible for shaping elite athletes’ sporting environments enact safe sport in everyday practice and engage with the organisational and sport-cultural conditions that shape these efforts. This represents an important public health knowledge gap because creating sporting environments that prevent harm and promote health, welfare, and positive development requires attention not only to individual behaviour, but also to the organisational and cultural conditions within which sport participation takes place. Examining how sport personnel navigate these conditions may help explain why harmful practices may continue to be reproduced despite increasing awareness of safe sport and the availability of endorsed safe sport policies.

The present study examines how safe sport is understood as a normative, organisational, and sociocultural practice within everyday sport settings. Understanding these processes requires a theoretical perspective capable of examining how individual actions are shaped by, and in turn shape, organisational and cultural conditions. We therefore draw on Strong Structuration Theory (SST) [37], which examines the dynamic interplay between agency and structure. More specifically, we investigate how sport personnel in elite athletics understand and enact safe sport and how these understandings and practices are shaped by interaction between their internal structures (e.g., beliefs, values, and knowledge) and the external structures within which they operate. In doing so, the study advances understanding of safe sport as a structurally embedded public health practice and identifies organisational conditions that may strengthen or constrain prevention capacity.

The specific research questions were:

- How do sport personnel in Swedish elite athletics understand and enact safe sport within their everyday practice?
- How do external structural conditions shape, enable, and constrain sport personnel’s understandings and enactments of safe sport?

## Theoretical framework

While the interplay between agency and social structure has been extensively examined and discussed within sociology [38]. Strong Structuration Theory (SST) [37] has primarily been associated with research on organisational change and management [39, 40]. SST offers a framework for examining the recursive interplay between agency and structure within situated social contexts. SST was considered particularly suitable for the present study because it provides an explicit analytical framework for examining how internal structures, external structures, agency, and outcomes interact in situated organisational contexts. Agency is understood as actors’ capacity to draw upon their knowledgeability and available resources to make a difference in practice, while structures consist of the rules and resources that both enable and constrain action. Through this ongoing interaction, actors reproduce or transform social structures in which they are embedded [37, 40]. Although still relatively sparsely utilised in sport research, SST has increasingly been applied as a theoretical framework to understand the dynamic between participants and sport environments across diverse settings and populations. Studies have explored topics such as African Americans’ involvement in basketball [41], the recursive relationship between agency and structure in sustained participation among youth sport male college [42], how social structures and cultural discourses constrain and enable agency among girls in community sport programmes [43], and how retiring professional rugby players and their significant others navigate the transition out of elite sport [44]. Collectively, these studies suggest the value of SST for understanding how participants in sport both shape and are shaped by the social structures in which they are embedded. SST also has growing relevance in health services and public health research because it can illuminate how health-related practices, policies and interventions are enabled, constrained, reproduced or transformed through the interplay among external structures, internal structures, active agency and outcomes [45]. Empirical applications of SST in health research have shown that interventions and policy initiatives do not operate independently in the contexts in which they are introduced. Professional values, organisational routines, material resources, policy expectations and local practices shape whether intended improvements are enacted in practice [46, 47]. These insights are relevant to safe sport because safeguarding policies, reporting channels, educational requirements, selection routines and accountability procedures may similarly function as external structures that shape sport personnel`s scope for action. At the same time, sport personnel’s values, experiences, professional knowledge and sport-specific dispositions may function as internal structures that influence how safe sport is interpreted and enacted.

Stones’ Quadripartite Nature of Structuration (QNS) model is an extension of the SST and provides a framework for analysing the interplay between agency and structure [37]. It conceptualises structuration as a diachronic process involving four interrelated elements: (a) external structures as conditions (institutional norms, legislation, economic and social systems that shape the actor’s scope of action and behavioural choices, though not necessarily explicitly recognised by the actor); (b) internal structures comprising the agent’s conjuncturally specific knowledge of external structures and dispositions (e.g., individual values, preferences, personality traits); (c) active agency, referring to concrete actions undertaken by the actor within a specific situational context; and (d) structural outcomes, which may be reproductive (i.e., reinforcing existing structures) or transformative (i.e., altering them) [37]. Thus, the QNS offers a framework to understand how agents reproduce or transform structures through both reflexive and unreflective practices and was employed as an overarching analytical framework guiding the analyses in the present study. In the present study, human behaviour is understood as embedded within a broader ecology of interconnected elements, including individual dispositions, interpersonal relationships, cultural norms, organisational structures, and wider societal influences. Accordingly, challenges are conceptualised not as isolated phenomena, but as emergent outcomes of dynamic interactions among these contextual factors [37, 48]. 

## Methods

### Study design

This study adopted a qualitative research design with reporting guided by the Consolidated Criteria for Reporting Qualitative Research (COREQ) standard [49] and the World Medical Association (WMA) Declaration of Helsinki - Ethical Principles for Medical Research Involving Human Subjects [50]. 

The study was informed by a social constructivist position [51]. Ontologically, the study assumes that organisational structures, practices and their consequences may exist independently of how they are perceived or understood, while recognising that meanings attached to safe sport are socially and contextually constructed through organisational, cultural and interpersonal processes and norms. Epistemologically, knowledge is understood as co-constructed through participants’ accounts and researchers’ interpretations, and as shaped by socially and culturally situated discourses and practices. Axiologically, we acknowledge that our values, experiences, and disciplinary backgrounds as researchers inevitably shape the research process and interpretation of data; these influences were addressed through ongoing reflexive engagement throughout the study (see Sect. 2.6) [51]. Consistent with this position, the aim was not to determine an objective truth about safe sport, but to explore how safe sport was understood and enacted within elite athletics environments.

### Study context and participants

Swedish elite athletics operates within a broader sociocultural context that is often characterised by relatively low power distance, a strong emphasis on equality, child rights, participatory decision-making, and institutional trust. The incorporation of the United Nations Convention on the Rights of the Child [52] into Swedish law in 2020 further reflects the societal importance attached to children’s rights and welfare. These societal values are reflected in Swedish educational and welfare systems and may shape expectations regarding leadership, athlete welfare, accountability, and appropriate conduct within sport settings. Consequently, understandings of safe sport in the present study should be interpreted in relation to this wider sociocultural context.

Swedish Athletics, governed by the Swedish Athletics Federation [53], maintains a strong international presence and encompasses multiple high-performance environments. In addition to individual elite clubs, these environments comprise seven specialised high-performance centres and seven accredited national elite sport high schools for able-bodied athletes, distributed nationwide, as well as national teams across age categories [53]. The high-performance centres, mandated by the Swedish Athletics Federation, aim to optimise conditions for elite-level development and training for athletes and coaches alike. They collaborate with national elite sport high schools, municipalities, universities, and other organisations, and employ elite-level coaches alongside multidisciplinary support staff (e.g., strength and conditioning trainers, testing personnel, physiotherapists, dietitians, sport psychologists). These high-performance centres also provide access to testing services and other elite sport-related resources [53]. National elite sport high schools constitute structured environments for talent development, targeting athletes aged 15–19. Accredited by the Swedish Sports Confederation and regulated by the National Board of Education, their core objective is to support dual career pathways by offering academic programs tailored to elite striving student-athletes’ needs, thereby mitigating potential tensions between educational responsibilities and athletic commitments [54–57]. Together, the high-performance centres, national elite sport high schools, and national team programs form the backbone of Sweden’s elite athletics infrastructure, providing strong athletic development conditions from the national junior elite level to the international senior elite level [53]. 

Participants in this study were purposively sampled to ensure representation across different athletic disciplines and organisational levels and within Swedish Athletics, including national elite sport high schools (*n* = 8) and high-performance centres and national team structures (*n* = 9). The study included 17 participants (8 women, 9 men; aged 35–66 years), all holding positions of responsibility, either on a voluntary basis or through part-time or full-time employment, for shaping elite athletes’ sporting environments within Swedish Athletics (e.g., national elite sport high school teachers or coaches, managers, and head coaches). These roles were embedded in formal structures operating across junior and senior elite levels, including national elite sport high schools, high-performance centres, and national teams. Teachers and coaches at national elite sport high schools typically worked within teams and played an active role in shaping athletes’ daily training and development within the Swedish national elite sport high school system. Participants holding positions of responsibility for high-performance centers were involved in decisions concerning resource allocation, organisational practices, and the coordination of support services, thereby influencing the conditions under which athletes trained and developed. National head coaches were responsible for national team programmes across different age groups. Collectively, these positions afforded participants both direct operational influence and indirect strategic influence over the development, organisation and safety of elite sport environments across organisational levels and age groups, ranging from junior to senior elite sport within the Swedish elite athletics system. As such, participants were well positioned to provide insights about safe sport in relation to the study aim and research questions.

Participants were recruited through contact with national elite sport high schools, high-performance centres, and national teams, with individuals holding responsibilities for the creation of sport environments invited to serve as interview participants. All invited sport settings, except for one national elite sport high school which declined due to limited time availability, accepted participation and were represented by one or more responsible sport personnel for sport environments under study. Several participants held multiple roles spanning different organisational levels, such as coaching both junior and senior athletes or combining responsibilities within clubs, national elite sport high schools, high-performance centres, and national teams. Given that the community of sport personnel in positions of responsibility for elite athlete development environments within Swedish athletics is relatively small and that individual identities may therefore be readily identifiable, we deliberately chose on ethical grounds to refrain from providing detailed participant information, as such details could compromise participant anonymity.

### Interview guide and data collection

We collected data using a semi-structured interview guide developed for this project [58], comprising open-ended questions allowing flexibility for follow-up and elaboration. The interview guide was developed to facilitate participants’ own exploration of their experiences, with probes used to clarify, deepen, and contextualise issues during the interview. This enabled participants to introduce and elaborate on what they considered meaningful in relation to their sporting context. Consequently, the interviews remained grounded in participants´ lived experiences to generate rich, in-depth perspectives, while the interview guide helped maintain consistency across interviews. The full interview guide, not previously published, is available as Supplementary File 1. All participants provided written informed consent before participation. All interviews were performed by the first author in Swedish, online via Zoom. Each interview began with general background questions (i.e., percentage of working time devoted to the current role within the athlete development environment and other sport related positions of responsibility, professional background within elite sport, experiences working with elite athletes at current performance level (junior/senior) and a brief outline of their professional philosophy and approach to athlete development). The total interview length ranged from 35 to 98 min (average: 55 min).

This study focuses on the first section of the interview guide, which explored participants’ understandings and experiences of safe sport through open-ended questions concerning the meaning of safe sport, perceptions of safety within their sporting environment, and factors contributing to safe and unsafe sport environments. Example questions included: *“What comes to mind when you hear the term safe sporting environments?”* and *“Are there aspects of the current sporting environment that you believe contribute to making it unsafe for athletes?”* The full interview guide is provided in Supplementary File 1.

### Data analysis and rigour

We analysed our data by using reflexive thematic analysis as outlined by Braun and Clarke [59, 60]. All interviews were manually transcribed verbatim. The first author conducted the initial familiarisation with the data and generated preliminary codes inductively. SST and the QNS framework [37] were not used to generate initial codes, but informed the organisation, interpretation and refinement of themes and subthemes during later interpretive stages of the analysis. Themes were subsequently reviewed, discussed and abductively refined in collaboration with the second and third authors to enhance analytical depth, coherence and consistency in interpretations. After completion of all coding, theme development, interpretation, and analytical decision-making, Copilot Premium, provided within Linköping University’s secure digital environment, was used to review the written presentation of the findings, identify potential overlaps, inconsistencies, or areas where the presentation could be clarified, and assist with the translation of selected quotations. No interview transcripts, audio recordings, or directly identifying participant information were entered into Copilot. Copilot was not used for coding, theme development, interpretation, or analytical decision-making. Final themes and interpretations were developed through negotiated reflexive dialogue. Where differences remained between authors’ interpretations, these informed refinements to themes and analytic claims. All translations of quotations were manually reviewed by the first author, who retained responsibility for the final wording and preservation of meaning. Minor editorial adjustments were made during the translation from spoken Swedish to written English to improve readability while preserving participants’ intended meaning.

Methodological integrity was pursued reflexively throughout the research process, using Tracy’s [61] criteria for qualitative quality as a broad sensitising principle. Credibility was supported through sustained engagement with the data, contextual familiarity with Swedish elite sport, and the use of illustrative quotations. Meaningful coherence was addressed by maintaining alignment between the research questions and the study’s ontological, epistemological, axiological and reflexive foundations. Purposive sampling across organisational levels within Swedish athletics provided sufficient information power to address the study aim [62]. We used Braun and Clarke’s 15-point checklist and the Reflexive Thematic Analysis Reporting Guidelines (RTARG) as reflexive aids to support methodological congruence, thematic coherence and reporting transparency [60]. 

### Reflexivity

As three authors with different disciplinary, professional and sporting backgrounds, we are positioned with both insider and outsider perspectives in relation to the elite sport context examined in this study. The first author (CL) is a researcher in psychology and sport sciences, a licensed psychotherapist, and an experienced sport psychology consultant in Swedish elite sport, including athletics, but has no personal background as an athlete or coach in athletics. This position may have facilitated familiarity with elite sport practices, organisational conditions and the language and norms of high-performance sport during data collection and analysis, thereby supporting contextual understanding and interpretation of participants’ accounts. At the same time, the first author’s prolonged engagement and familiarity with elite sport environments may also contribute to a degree of adaptation to assumptions, norms, and practices (e.g., cultural expectations or beliefs about training practices, sacrifices necessary to reach international success, and performance-oriented norms) often taken for granted or endorsed within high-performance sport. Engagement with co-authors, where we all were acting as critical friends, actively questioning assumptions, challenging interpretations, and offering alternative perspectives, was therefore viewed as important to the interpretative process. The second author (MJ) is a physician and researcher with experience as an athlete, coach and judge in another sport. This author’s position may have facilitated cross-sport reflections while drawing our attention to similarities between athletics and other sports, creating opportunities to critically examine sport-specific norms and assumptions that, from an insider perspective within a particular sport culture, may otherwise be perceived as natural, inevitable, or self-evident. The third author (LK) is a physician and researcher with expertise in child and adolescent psychiatry, safeguarding and violence prevention, but more limited experience of elite sport. This latter perspective may have directed attention towards issues of vulnerability, dependency, and organisational responses to harm, while potentially foregrounding safeguarding concerns. We treated these subjectivities as analytic resources rather than sources of bias to be eliminated. Through continuous reflexive dialogue, we used our differing backgrounds and perspectives to identify taken-for-granted assumptions and critically consider how our views regarding athlete welfare and safety, safeguarding, and institutional accountability might shape data interpretation and the construction of findings. Power relations and researcher positioning were also continuously considered. We critically reflected during our analysis on how our professional backgrounds, positions and varying degrees of familiarity with elite sport influenced interactions with participants, interpretations and knowledge production.

## Results

Using Stones’ QNS framework [37] as an interpretive analytical framework, we developed three overarching themes and associated subthemes. Together, these themes illustrate how internal structures, external structures, active agency, and structural outcomes interacted in sport personnel’s understandings and enactments of safe sport within Swedish elite athletics (Table 1). Theme 1 primarily concerns the interaction between internal and external structures, Theme 2 focuses on active agency, and Theme 3 highlights structural outcomes, including both the reproduction and transformation of existing practices.

**Table 1:** Summary of themes and subthemes illustrating how safe sport was understood, enacted, reproduced, and transformed within Swedish elite athletics

Theme	SST focus	Subthemes	Public health implication
Negotiating safe sport	Internal and external structures	Holistic safe sport	Safe sport depends on how sport personnel interpret and prioritise health, welfare and the prevention of harm within different institutional settings
		Temporal change and the reconfiguration of norms	
		Resources to provide safe environments within and beyond the sport context	
Enacting safe sport	Active agency	Collaborative agency	Everyday practices including collaboration, role-modelling and early intervention, support prevention within sport settings.
		Micro-level intervention	
Constraints and opportunities for safe sport	Structural outcomes: reproductive or transformative outcomes	Solitary coaching, status hierarchies and power asymmetries	Informal hierarchies and performance cultures may reproduce exposure to harm, while formalised procedures can counteract constraints and strengthen accountability and organisational prevention capacity.
		Institutional silence and informal mechanisms	
		Performance norms justifying normalisation of harmful practices	
		Fragmented and fickle accountability	
		Institutionalisation of safeguarding practices	

### Theme 1: negotiating safe sport

Theme 1 captures how sport personnel understood and interpreted safe sport through the interaction between internal and external structures. It comprises three interrelated subthemes: 3.1.1 Holistic safe sport, 3.1.2 Temporal change and the reconfiguration of norms, and 3.1.3 Resources to provide safe environments within and beyond the sport context. Swedish elite athletics sport personnel’s explanations of safe sport were commonly tied to the institutional setting they described, signalling that their understandings of safe sport were at least partly context‑dependent. They drew upon internal structures to conceptualise and enact safe sport while these enactments were simultaneously shaped, enabled, and constrained by external structural conditions. Throughout this process, sport personnel engaged in an ongoing negotiation between internal and external structures in constructing safe sport.

#### Holistic safe sport

Internal structures, such as personal norms and values grounded in holistic, person-centered philosophies, shaped how the sport personnel interpreted safe sport and negotiated with external structures. Participants frequently described safe sport as a shared value orientation that should guide everyday interactions, decision-making, and responsibilities across the sport system. In this shared value orientation, the responsibility for “safe athletics” was embedded across all roles in the sport system, constituting an aspect of everyday professional practice rather than being delegated to a single position. Such a shared commitment was perceived to strengthen collective accountability:

There is a position at the athletics federation that’s called ‘safe athletics’ or something. For me, I find it hard to understand why one person works with safe athletics. I think it should be something that’s part of every position. Everyone should work with safe athletics, right? (Interviewee 14, personnel at high-performance center or national team)

Attending to athletes’ perceptions of safety and minimising experiences of unsafety in a non-judgemental manner were described as central components:

A great part of feeling safe is about not feeling unsafe. In order not to feel unsafe, one needs a welcoming atmosphere and assurance of not being judged. That I, as a coach, listen carefully and, when I hear how someone experiences something, try to respect that this is how this person perceives it. (Interviewee 13, personnel at high-performance center or national team)

Creating supportive and safe environments, in which both personal and athletic development are nurtured, was positioned as a central aspect of safe sport across interconnected sport settings (training, academic, and residential). While performance and competitive success were acknowledged as salient within Swedish elite athletics, sport personnel described prioritising athletes’ wellbeing, personal growth, and long-term development over immediate sporting outcomes. In this sense, success was framed in relation to individual value orientations embedded in professional philosophies:

Care may sound a bit unusual in this context, but for me it is about seeing the whole person, or the whole athlete. It is exciting when things go well and some may aspire to reach the Olympics, but for me, that is not the ultimate goal. (Interviewee 3, personnel at national elite sport high school)

In national elite sport high school contexts, where dual career pathways are structurally embedded in daily practice, sport personnel further linked safe sport to a long-term developmental perspective that included encouraging student-athletes to envision futures beyond elite sport:

We work according to the principle of “the whole person, all the way”. We talk a lot about dual careers. What should you do after sport high school? There are opportunities to study at university and remain in the same environment with the same coach. Not trying to rush anything. Instead aiming for a long-term plan for these impatient young people, especially in the junior age groups, where there is a lot of pressure to compete at championships. (Interviewee 6, personnel at national elite sport high school)

Cultural variation was further noted in relation to professional styles and expectations of appropriate conduct. Respondents emphasised that norms of safe sport, i.e., what counts as “safe” or “appropriate”, may differ across national and cultural contexts:

For example, I have colleagues in [European country] where one of them took over a relay team that had previously had a rather authoritarian coach. When the new coach tried to work in a more open way, it created confusion: “Just tell us what to do!” They were not used to that. They were used to the coach giving explicit instructions. (Interviewee 17, personnel at high-performance center or national team)

#### Temporal change and the reconfiguration of norms

Sport personnel commonly understood athletics’ current safe sport status through their own experiences. Participants reflected on a positive change over time, noting substantial improvements in safety compared to earlier decades. They described how normative expectations within the athletics field have evolved, gradually reconfiguring what is considered acceptable practice:

In the late 90s and up until now… Then you’d hear stories from even earlier, and it wasn’t a safe environment. As a rookie [in the national team], you were more afraid of the initiation rituals than of participating in front of over 50,000 spectators or worrying about how many points you’d score or whether you might fail athletically. You were more concerned about how the initiation rituals would unfold and what you’d be forced to go through. To me that’s a pretty clear example of how things have evolved over these years. Now I think most people find that environment quite fun. (Interviewee 13, personnel at high-performance center or national team)

While participants acknowledged a shift towards lower tolerance in athletics of harmful practices and greater awareness of athlete welfare and safe sport principles, they emphasised that discursive change alone is insufficient. Instead, concrete action is required to translate awareness into practice:

Before, there was a lot of talk. There were lectures and workshops on safe sport and other things, but it was just talk; nothing was done. People knew these guys were doing this, but nothing was done about it. Only now has something started to happen, and I’m very glad about that. I think even more will need to happen to tidy things up a bit more in club life and elite sport, so to speak. (Interviewee 1, personnel at national elite sport high school)

Taken together, these accounts suggest that shifts in safe sport norms have occurred incrementally over time, reflecting a gradual reconfiguration of what is considered acceptable practice within the sport system.

#### Resources to provide safe environments within and beyond the sport context

External structures like the specific sport context and available material resources shaped what was seen as possible, necessary, and legitimate in practice regarding safe sport. For example, proper physical training and competition environments were noted as a prerequisite for safety:

It’s also about what kind of equipment we have, everything is about not getting injured during training. That’s also a safe environment. That we have good facilities that are healthy. That there isn’t bad air, poor ventilation, or bad tracks that could cause injury. Everything is really a safe environment”. (Interviewee 7, personnel at national elite sport high school)

Differences in organisational infrastructure across settings were repeatedly described in the interviews, particularly between national elite sport high schools and the broader sport community (i.e., sport clubs, high-performance centres, national teams). Safety‑related practices and routines were perceived by participants to have progressed more rapidly in adjacent societal domains (e.g., school) than within the broader sport community:

I think it has gone faster, like in the school world. And in some cases, like now, that it is starting to show results in club life and other areas that might be the last step. Maybe the last ones to do things. (Interviewee 1, personnel at national elite sport high school)

National elite sport high school settings were expressed to have most formalised safety structures, with higher standards, clearer guidelines, stricter requirements, and greater access to safety resources like student health services with qualified staff. By contrast, the broader sport community with sport clubs and national team settings, was perceived to rely more on voluntarism and individual responsibility:

The school is really great, they have such incredible resources. But if something like this happens at a championship or an international setting […] What resources do we have to act on it? (Interviewee 11, personnel at high-performance center or national team)

Available resources and formal requirements perceived in sport environments also shaped the prioritisation of safe sport. Safe sport was perceived to be more likely to be foregrounded and routinised in the daily work if formally required and evaluated, like in national elite sport high schools, in contrast to ad hoc approaches more commonly adopted in sport clubs:

In school we have these requirements, it feels more like that… Or not requirements, but we have to work on it. Every year we have to work on values and safety, it comes up all the time. But in athletics, that doesn’t happen in my club. (Interviewee 3, personnel at national elite sport high school)

In contrast to national elite sport high schools and sport club settings, national team settings were described as episodic and characterised by varying team composition, which could hinder continuity and sustained contact among agents in the national team environment (e.g., athletes, coaches, support staff, entourage). Participants described this temporal and relational fragmentation in national team structures contributing to coordination challenges:

It’s the personal coaches and the athletes who do it and create the conditions for this group to be selected and perform well. But it’s a slightly different situation from having your coach with you all the time to not having your coach or the coach sitting in the stands and not being allowed into the national team’s area. (Interviewee 4, personnel at national elite sport high school)

### Theme 2: enacting safe sport

Theme 2 captures how sport personnel exercised agency through everyday practices within their organisational contexts. It comprises two subthemes: 3.2.1 Collaborative agency and 3.2.2 Micro-level intervention. External structures, including material resources, organisational contexts, governance arrangements and cross-institutional processes, conditioned the context of safe sport action. By drawing on their internal structures, sport personnel exercised agency through practices such as team-based coaching, and micro-level interventions such as role modelling. These practices illustrate how the notion of shared responsibility, described at a normative level in Theme 1, was enacted in situated coaching contexts.

#### Collaborative agency

Sport personnel enacted safe sport through team-based coaching practices underpinned by a shared commitment to long-term athlete safety. This was described as an important mechanism for cultivating environments which contributed to relational safety. Expanding coaching structures through collaboration was further perceived to increase athletes’ access to trusted relationships, thereby enhancing opportunities for disclosure when needed. Recruitment practices were also an active part of safe sport enactment. Rather than relying on informal networks or like-minded individuals, recruitment was framed by participants as intentional and value-driven, prioritising diversity, collaboration, and a shared commitment to safety. A balanced composition in terms of gender and generational diversity was perceived as contributing to a more inclusive climate and reducing potential power asymmetries:

That we have both male and female coaches […] I also think we mix old and young. […] I think that creates a very good sense of safety for us who work there. If it had only been men, things probably would have looked very different. (Interviewee 2, personnel at national elite sport high school)

#### Micro-level intervention

Sport personnel described engaging in ongoing efforts to shape everyday interactions through role-modelling and proactive intervention, with the aim of reinforcing norms related to safety, respect and inclusion. A recurring theme in the interviews was the emphasis on equality and role-model responsibility. Sport personnel who viewed themselves as embedded in a professional culture characterised by trust, humility, and open communication were considered better positioned to model and reproduce norms related to safety and belonging:

For me, it’s really important to have a safe coaching team that puts the team before the individual. We are humble, free from prestige, we share aspects of ourselves, and we model how we want the group to behave. (Interviewee 11, personnel at high-performance center or national team)

Participants highlighted that athletic success should not confer greater social value within training environments. Instead, all athletes were expected to be treated with equal respect regardless of performance level. Intentional efforts to organise training and social activities fostered group cohesion, which functioned as a protective factor against insecurity and social exclusion. Participants described shared training sessions across genders and performance levels as contributing to cohesion, fostering a sense of belonging, mutual respect, and appreciation. These relational dynamics were perceived to enhance both athlete wellbeing and performance:

Even when we look at perhaps the very best, why they succeed, it’s often that they talk about the others in the group. Those who never get to run a World Championship […] but who mean so incredibly much for their effort to keep things going. So the atmosphere is extremely important, whether you’re going to run a World Championship or the Olympics, or trying to get a place in a relay team at the Swedish Championships. (Interviewee 15, personnel at high-performance center or national team)

High-performing athletes were encouraged to be particularly attentive to their language and behaviour, given their influence on younger peers. Sport personnel actively addressed behaviours that crossed normative boundaries, aiming to maintain a respectful and psychologically safe climate. Early proactive intervention in everyday interactions, such as language use and peer dynamics, was framed as central to preventing the escalation of harmful practices:

You are probably familiar with the so-called broken window theory. […] The idea that even small issues should be addressed immediately. This applies to how we speak to each other. […] It starts with the coaches, how we behave toward one another and toward the athletes, and how we intervene when we notice something among them. (Interviewee 5, personnel at national elite sport high school)

### Theme 3: constraints and opportunities for safe sport

Theme 3 captures structural outcomes of safe sport practices, including both the reproduction of unsafe conditions and opportunities for transformation. It comprises five subthemes: 3.3.1 Solitary coaching, status hierarchies, and power asymmetries; 3.3.2 Institutional silence and informal mechanisms; 3.3.3 Performance norms justifying the normalisation of harmful practices; 3.3.4 Fragmented and fickle accountability; and 3.3.5 Institutionalisation of safeguarding practices. The agency of enactment described in Theme 2 was simultaneously constrained by entrenched status hierarchies, power asymmetries and performance-oriented norms within elite sport culture, which further contributed to the normalisation and reproduction of harmful practices and institutional silence. To counteract constraints and increase accountability, safeguarding practices needed to be embedded structurally within the athletics organisation.

#### Solitary coaching, status hierarchies, and power asymmetries

Participants described solitary coaching arrangements as a significant risk factor for unsafe sport environments, as they may reduce transparency and limit opportunities for oversight. This dynamic was described as potentially creating strong dependencies, rendering athletes vulnerable to accusations of disloyalty if they sought to switch coaches. They portrayed this issue as widely recognised, yet difficult to address, given the voluntary nature of sport organisations and the absence of formalised accountability structures:

Many of the coaches are volunteers, and these are volunteer-based organisations where it may not be entirely easy to navigate small, independent environments in which the broader community is not fully aware of what is happening. Much depends on the leaders, the coaches and trainers, and their actions to ensure that athletes and other coaches experience the environment as safe. (Interviewee 13, personnel at high-performance center or national team)

Problems associated with solitary coaching arrangements included tendencies to treat athletes as possessions and to isolate them, thereby shifting the focus from the athlete’s development towards advancing the coach’s own career. This was justified through a performance demand culture:

It’s great that people volunteer, but there has been no control over who they are. What kind of people come in. As long as they deliver results and make it to the Olympics or World Championships, people have been satisfied. It doesn’t matter what they said or did; they’ve been allowed to do exactly as they please as long as the results come. (Interviewee 2, personnel at national elite sport high school)

Participants suggested that these risks may be intensified by the power asymmetries embedded in coach–athlete relationships, particularly in the absence of formal regulation regarding appropriate conduct. Respondents noted that such dependencies could create conditions for exploitation, with clubs at times rationalising problematic relationships. One participant illustrated this concern through a hypothetical example of a coach-athlete relationship in the Swedish context, where the legal age of consent is 15 years, arguing that Swedish sport lacks clear regulations governing relationships characterised by dependency and power imbalances. As a result, sport clubs may at times minimise or rationalise situations that remain problematic due to significant age differences and clear power asymmetries:

It’s a position of dependency […] [If] I’m a single 50-year-old and I really like this person who is 15, then the club can turn it into “well, you’re both adults.” You can actually get something like that in return. (Interviewee 2, personnel at national elite sport high school)

#### Institutional silence and informal mechanisms

At a broader institutional level, beyond individual coach–athlete relations, a recurring structural constraint concerned the association of elite coaching with status, which was primarily linked to having contributed to athlete success rather than to formal qualifications. This reflects how external structures, such as the absence of clear credentialing standards, can enable informal pathways to authority, thereby reinforcing performance-based norms over formal educational or professional requirements:

It would be much easier if we had a clear standard such as: “To be an elite coach in Swedish athletics, you must hold a university degree, and then all elite coaches meet that criterion.” As it stands, it is very difficult to define. Someone might simply get lucky by discovering a talented athlete and, if they do not limit that talent too much, they are suddenly regarded as an elite coach. Even if they may not have substantial knowledge. (Interviewee 14, personnel at high-performance center or national team)

Participants described environments perceived as both healthy and unhealthy, including cases of inappropriate coach–athlete relationships. High-status coaches could be perceived as institutionally protected, shielded by a culture of silence and network-based loyalty. This illustrates how interpretive schemes and normative expectations within small, tightly knit communities may pose a risk of constraining corrective action and sustain problematic practices:

There is, in fact, a culture of silence. Coaching is a rather small circle. How many coaches are we? Perhaps around 50 employed in athletics in some capacity, maybe a few more if part-time positions are included, but those who make a living as athletics coaches form a very small group. Moreover, there has always been a degree of nepotism in athletics, who knows whom, who is friends with whom, and this has fluctuated over time. (Interviewee 14, personnel at high-performance center or national team)

#### Performance norms justifying normalisation of harmful practices

Participants described athletics as an inherently performance-driven sport due to its highly measurable nature, which was perceived as giving rise to strong performance norms and an embedded expectation to continuously improve:

But if you look at athletics in general, it is an extremely performance-based sport where everything can be measured down to thousandths of a second and millimetres. So, in a way, you have to accept that if you want to enter the world of athletics, everything is measurable. And already there, a certain kind of performance pressure is created. You simply can’t escape the fact that this is a results-based sport. (Interviewee 6, personnel at national elite sport high school)

Although social interactions within training groups were generally described as supportive and encouraging, sport personnel expressed that athletes often internalised performance norms and associated their own value and acceptance with demonstrating progress and achieving results. As athletes progress through the elite sport pathway, achievement was perceived to displace athletes’ joy and curiosity, reflecting a transition towards results-driven interpretive schemes:

Among younger athletes, I see much more joy and curiosity. That’s what has been so fantastic about these talents. There is so much energy and enthusiasm. Somehow, that gets blunted as they move into the senior level and become more focused on goals and performance. (Interviewee 13, personnel at high-performance center or national team)

While sport personnel actively sought to regulate behaviour and promote safe environments (Theme 2), these findings indicate that broader sport-cultural norms may simultaneously counteract such efforts. Several sport personnel described attempts to broaden athletes’ interpretive frameworks by encouraging them to value aspects of development that are less easily quantified, such as technical refinement, recovery, nutrition, sleep, and academic work, yet remain essential for long-term wellbeing. Despite these efforts, the expectation to demonstrate continual improvement was perceived to generate pressure, which was thought to lead to performance anxiety and feelings of inadequacy:

I think they burden themselves with it… It turns into performance anxiety. Some of them are actually held back in their development because they are afraid they won’t improve. And that, in itself, can create an insecure environment. Walking around with that kind of pressure doesn’t contribute to a healthy climate, even if they are supportive and encouraging towards each other. (Interviewee 9, personnel at high-performance center or national team)

Participants also described the risk of a “bullying culture” emerging when harmful behaviours become normalised or dismissed as “just the way it is.” Such normalisation was viewed as incompatible with the goal of creating safe environments. These accounts suggest that broader sport-cultural norms and expectations may shape understandings of acceptable conduct within elite sport. Concerns were expressed regarding the vulnerability of young athletes, especially those who had moved away from home to pursue sport and lacked close parental support. Gender norms were further highlighted as potentially obscuring boys’ vulnerability in psychologically harsh environments, where expectations of toughness may discourage expressions of distress and help-seeking:

It may sound surprising, but young boys’ emotions are not as resilient as one might assume, especially when they are placed alone in a situation. In those moments, they can be just as fragile as the girls. (Interviewee 2, personnel at national elite sport high school)

Together, these accounts suggest that broader cultural expectations surrounding acceptable behaviour, vulnerability, and resilience may contribute to the reproduction of unsafe practices even when such practices are recognised as problematic.

Several sport personnel also raised concerns that everyday interactions can contribute to exclusion or psychological pressure, even in the absence of overt misconduct. In some cases, such behaviours were described as being normalised or rationalised as part of competitive sport, contributing to the reproduction of performance-oriented norms and expectations within the sporting environment. It was noted that athletes themselves may, at times, also actively seek out or remain in such environments, thereby demonstrating compliance with or tolerance of problematic behaviours when they are perceived as instrumental to achieving success. When athletes orient their actions toward performance-oriented norms, unsafe practices may be reproduced:

I believe that many athletes want to achieve very high results and may either overlook or turn a blind eye to certain things to reach the best possible outcome… They feel that “to go all the way, maybe it’s worth dealing with people who probably shouldn’t be involved.” (Interviewee 1, personnel at national elite sport high school).

#### Fragmented and fickle accountability

Voluntary and contractual employment arrangements were reported to create ambiguity in the allocation of responsibility when incidents occur. The national federation was viewed as offering limited, poorly communicated formal or clear safety guidelines (such as crisis management resources), which undermined preparedness and risked producing insecurity:

When a newly formed national team staff steps in and there’s a new coaching team, it’s kind of… It’s just expected that we should say ‘yes, here we go!’ […] What’s important if something happens? How do we handle it? Where are the quick guides for this? Who do we turn to? (Interviewee 11, personnel at high-performance center or national team)

Sport personnel discussing safe sport from a national team perspective also related safe sport to cross‑national cultural differences and security risks at certain international events (e.g., contexts marked by high criminality or political conflict). In such conjunctures, sport personnel prioritised getting access to reliable information from public agencies (e.g., Ministry for Foreign Affairs) and the establishment of secure conditions for the team’s participation, also aiming to create a sense of calm so athletes could focus on performance:

I have focused immensely on mitigating the risks, purely safety risks. So that the athletes can focus on their performance and translate what they have practiced on at home and in many competitions they are used to into this very pressured or stress-filled situation. […] So if I, in the coaching team and the medical team, can help to be that sense of safety. That is our most important mission (Interviewee 11, personnel at high-performance center or national team).

Practical governance processes, like transparent qualification criteria and a clearly defined, consistently enacted selection process for championships, were identified as central in elite sport contexts to fostering trust and perceived safety. Ambiguity or inconsistency in the application of selection criteria was mentioned, for example, when subject to cross-institutional negotiation (e.g., between national sport federations and higher-level governing bodies), which was then associated with a fickle system and perceived unsafety:

We’ve had Swedish Athletics and the Swedish Olympic Committee having different views on… Well, selection. And even athletes taking it to CAS [the Court of Arbitration for Sport], against the Swedish Olympic Committee. You could say that in elite sport, that’s also a kind of stress. That I’ve done my best, I’ve delivered what’s required, but I still don’t get to go to what I dream about. To a championship. So that’s also a kind of unsafety. (Interviewee 17, personnel at high-performance center or national team)

#### Institutionalisation of safeguarding practices

Several sport personnel underscored the necessity of structurally embedding safeguarding practices across the athletics organisation. The adoption of a zero-tolerance approach in sport organisations to unsafe practices and inappropriate behaviours was identified as critical for fostering a culture of accountability and safety. However, participants stressed that such an approach must be accompanied by tangible consequences to ensure that individuals feel secure in reporting incidents. In the absence of clear repercussions, the perceived risk of speaking up was considered substantial:

They now know that “this is not okay,” and that it will be taken further, and they know something will happen. Before, I think they didn’t trust that anything would happen anyway. That it didn’t matter, it was better not to say anything because then you’d only expose yourself. Nothing would happen. But now maybe they take that risk because they know something will happen, and that’s where we need to get to. (Interviewee 2, personnel at national elite sport high school)

Participants emphasised the importance of implementing safety surveys, whistleblowing mechanisms, clear reporting procedures, and action plans to facilitate the reporting of unsafe situations or irregularities:

What’s good are these whistleblower channels that have emerged in recent years. At least, they might open the door for someone to dare to step in and pull the brake. Hopefully. (Interviewee 1, personnel at national elite sport high school)

Uncertainty regarding available courses of action and appropriate channels for response was considered a constraint for action:

I’m absolutely no specialist in these issues, you know, and that’s not why I’m in this job. It would have been so much better for both parties, and honestly easier for me, if I could just hand it over… I mean, I observe things and keep my eyes open in national team settings, at meetings, and in all sorts of contexts, but if I see something, I want to be able to pass it on. (Interviewee 14, personnel at high-performance center or national team)

Taken together, these findings illustrate how the institutionalisation of safeguarding practices could provide sport personnel with clearer modalities for action, reducing uncertainty and enabling more consistent and accountable responses to unsafe situations.

## Discussion

Using Strong Structuration Theory (SST) as an analytic framework [37], this study explored how sport personnel with responsibilities for sport environments in Swedish elite athletics understood and enacted safe sport, and how these enactments were shaped, constrained or enabled by structural conditions. The findings show that safe sport was widely endorsed as a holistic, value-driven ideal, but that its enactment was shaped by organisational resources, accountability arrangements and performance-oriented norms. While previous research has highlighted the risk of gaps between safe sport policies and practice [7, 11, 12, 20, 63, 64], the present study contributes to identifying organisational conditions that may help explain such gaps.

One main contribution of this study is to show how safe sport is shaped through everyday interactions between sport personnel, organisational conditions, and broader sport-cultural norms. Sport personnel in this study did not simply implement or reject safe sport principles; rather, they interpreted, negotiated and enacted them through situated knowledge, professional dispositions, organisational conditions and sport-specific norms. Safe sport was described by participants both as a set of personal values and responsibilities and as a set of organisational expectations, policies and procedures. In SST terms, these understandings may be viewed as reflecting both internal structures (e.g., values and responsibilities) and external structures (e.g., policies, procedures and organisational expectations) [37, 45–47]. Where these external structures were perceived as fragmented, weak, or poorly communicated, sport personnel’s capacity for preventive action remained constrained, even when their personal values aligned with safe sport principles.

### Organisational conditions for safe sport as a public health practice

Our findings support the view that safe sport should be understood not only as a safeguarding or compliance issue, but as a broader settings-based public health practice. In this regard, safeguarding is best understood as one component of safe sport rather than an equivalent concept. However, this does not imply that safe sport replaces more specific concepts such as safeguarding, injury prevention, or health promotion. Rather, safe sport provides a broader organisational and sociocultural framework within which these more specific practices and interventions may be understood and coordinated. Sport personnel’s understanding of safe sport in broad and holistic terms aligns with contemporary safe sport research that extends beyond the prevention of interpersonal violence to include wider concerns such as injury prevention, mental health, equity, accountability and human rights [9, 12–14]. However, the findings also show that safe sport was interpreted differently across institutional contexts. The holistic and participatory understandings of safe sport identified in this study may also partly reflect broader Swedish societal norms emphasising equality, child rights and participatory decision-making. National elite sport high schools were described by participants in this study as having relatively formalised structures whereas clubs and national team settings were described as more fragmented and dependent on individual sport personnel. This variation is important from a public health perspective because prevention requires not only shared values but also organisational conditions that support the implementation of safeguarding practices as well as broader safe sport efforts. Safe sport thus depended on both individual commitment and organisational structures that enabled action. Participants also contributed to reproducing and, at times, reshaping these practices through their everyday decisions. This finding resonates with SST-informed public health and health services research [45–47]. For example, SST-based studies of healthcare interventions have shown that formally introduced policies and programmes are not implemented uniformly, but are shaped by local organisational routines, professional norms, available resources, and actors’ interpretations of their roles [46, 47]. Similarly, the present findings suggest that safe sport principles may be widely endorsed but enacted differently depending on the organisational conditions within which sport personnel operate. As with healthcare interventions that may be formally introduced but unevenly adopted, safe sport policies may only become meaningful in practice when they are translated into everyday actions, organisational routines, and appropriate resources. The findings suggest that safe sport may therefore be understood as a practice that is continuously produced through everyday interactions between sport personnel, athletes, organisations, policies and material or financial conditions [6, 17]. 

### Why awareness of safe sport is insufficient

Although our findings indicate that awareness of safe sport had increased, for example, through lectures, workshops and discussions in athletics, participants’ accounts also illustrated how harmful practices could still be tolerated or left unaddressed in practice. Safe sport norms in athletics seemed to have shifted incrementally over time, reflecting a gradual reconfiguration of what is considered acceptable practice within the sport system. At the same time, sport personnel also described the persistence of problematic norms highlighted in Theme 3, indicating that cultural transformation is uneven and only partially consolidated. Performance-oriented expectations appeared to obscure what was conceptualised as safe sport and which norms were justified and normalised. This aligns with previous research showing that unsafe sport environments are often characterised by performance cultures that prioritise short-term success over athletes’ welfare, long-term health, and development, also shaping what sport personnel, athletes and organisations perceive as normal, necessary or acceptable [5, 6, 9, 65]. Such performance-oriented norms have long been associated with sporting cultures that valorise endurance, sacrifice and practices such as “playing hurt” [66, 67]. From an SST perspective, however, playing hurt is not simply an individual choice but an example of how agency and structure interact in the reproduction of sport norms. Athletes may actively choose to train and compete despite pain or injury, yet these choices are made within organisational and cultural contexts that reward sacrifice, normalise risk-taking and associate toughness with commitment and success. As demonstrated by Ryou and Lee [68], professional combat athletes frequently rationalised playing hurt through an implicit belief that doing so would yield valued rewards, including cultural, social, economic and performance capital, thereby contributing to the ongoing reproduction of risk, pain and injury cultures through their own actions [68]. In this way, increased awareness of safe sport may coexist with the continued enactment of unsafe practices when the broader structures that legitimise such behaviours remain largely unchanged.

Processes of socialisation within sport may begin early in life and shape how individuals interpret and respond to behaviors within their environment [69]. As athletes transition into leadership roles, such as coaches, judges, or administrators, they may carry forward internalised norms and expectations, thereby reinforcing shared understandings within the sporting community. These processes are further sustained through group dynamics, in which belonging, identity formation, and emotional investment are closely tied to conformity with established norms and values [15, 70]. Membership in the ‘sport community’ thus entails alignment with the values and qualities associated with sport itself. Moreover, the enduring positive social image of sport, as inherently beneficial, developmental, and morally sound, may obscure the recognition of harmful practices and delay critical scrutiny [27, 71]. Our findings suggest that sport personnel appeared to draw upon safe sport principles as a normative framework through which athlete welfare, appropriate conduct and organisational responsibility were interpreted. However, as in previous SST-informed analyses, a normative framework can shape how actors understand desired outcomes yet still fail to provide resources when needed [46]. From an SST perspective, these findings suggest that bullying cultures and gendered expectations may function as external structures that shape understandings of acceptable conduct and vulnerability within elite sport. When internalised by athletes and sport personnel, such norms may contribute to the reproduction of unsafe practices through everyday interactions, even when they are recognised as problematic.

The findings further suggest that silence and inaction may be shaped by sport traditions and informal power structures that constrain participants’ agency to act beyond micro-level practices, rather than simply reflecting individual failures of courage, knowledge, or commitment. This is important because prevention strategies that focus on only individual awareness may overestimate what sport personnel can do when external structures remain ambiguous and governed based on unspoken or unquestioned traditions [65]. In SST terms, sport personnel’s agency is enabled and constrained by their knowledge of the strategic terrain; what procedures exist, what consequences are likely, who has authority, and whether reporting is expected to lead to meaningful action [37]. Awareness may change internal structures, such as values and interpretations, but transformative change also requires external structures that redistribute responsibility, clarify expectations and make actions possible. Without such structures, safe sport risks remain a normative aspiration rather than an enacted public health practice.

### Implications for public health and sport governance

Our findings, with participant accounts analysed through an SST framework, suggest that safe sport depends on the alignment between internal structures and the broader organisational conditions that enable or constrain action. As such, the findings have important implications for both public health and sport governance. The findings suggest that safe sport cannot be achieved solely through efforts aimed at preventing or responding to individual incidents of harm. Rather, safe sport also requires attention to the broader organisational and sociocultural environment in which sport is conducted. Interventions implemented within sport organisations should address both harmful behaviours and the organisational conditions that allow such behaviours to persist. First, prevention efforts are unlikely to be effective when responsibility remains primarily located at the individual level rather than being embedded within organisational systems and supported by structures and resources that foster safe sport practices. For example, participants described how national elite sport high schools benefited from more formalised structures, including routine work on values, safety, and access to student health services (see Theme 3.1.3). These findings suggest that safe sport may be strengthened when safeguarding, welfare promotion, mental health support, and athlete development are embedded within existing organisational systems rather than treated as stand-alone compliance activities. Second, safe sport governance needs to attend to how accountability is distributed along an athlete’s full sport career trajectory (e.g., sport clubs, national elite sport high schools, high-performance centres, national teams). Identifying and prohibiting misconduct is, however, insufficient if the “sporting world” does not also institutionalise positive norms, desired practices and evidence-informed prevention strategies [12–14, 72]. Third, safe sport should be approached as a complex or “wicked” public health challenge [73]. Interventions in sport systems are unlikely to produce linear or universal outcomes when interacting with existing structural conditions. Frameworks developed outside sport, such as the WHO INSPIRE framework for preventing violence against children, may offer useful guidance [72]. While similar approaches have also been proposed in safe sport research, the field continues to lack consistent conceptual definitions which complicates efforts to assess prevalence, examine associations with outcomes, and design or evaluate proposed interventions or safe sport practices. Establishing clearer conceptual boundaries for safe sport is therefore essential for strengthening both research and practice and supporting more robust evidence-based decision-making within sport systems.

### Strengths and limitations

The sport personnel included in this study were drawn from Swedish athletics and were sampled to provide insight into high-performance contexts across multiple structural levels. Their cultural and educational backgrounds may have shaped their perspectives. Given that constructs such as safe sport are inherently shaped by cultural, social, and contextual conditions, the findings should therefore be interpreted in relation to their specific context rather than as universally applicable. While this study was situated in Swedish elite athletics, it is reasonable to assume that the findings speak to broader international public health and sport governance challenges. Many sport systems internationally combine formal safeguarding policies with decentralised clubs, voluntary positions, performance-based status hierarchies and episodic national team structures [74–76]. The study therefore provides conceptual insights into how safe sport principles may be broadly supported within sport systems while their implementation remains uneven across organisational contexts. The findings also highlight the importance of identifying factors that both reproduce unsafe sport practices and enable shifts towards safer practices. This includes not only safeguarding measures aimed at preventing and responding to harm, but also broader organisational and sociocultural conditions that support safe, healthy, and inclusive sport environments. Such insights are critical for enhancing athlete safety, health, and performance, supporting sport organisations (e.g., clubs, federations, and governing bodies), and reinforcing the ethical foundations of the sporting community in general.

### Conclusions

The findings indicate that sport personnel generally understood safe sport as extending beyond safeguarding and the prevention of harm to include broader organisational and sociocultural conditions that support athlete welfare. The results also point to a progressive but uneven transformation of safe sport in Swedish elite athletics. While awareness and norms have shifted, structural conditions continue to reproduce harmful practices. Consistent with SST and a settings-based public health perspective, advancing safe sport is likely to require organisational structures that strengthen prevention capacity, support accountability and enable sport personnel to translate safe sport principles into everyday practice.

## Supplementary Information

Supplementary Material 1.

## Data Availability

Availability of data and materials: The datasets generated and/or analysed during the current study are not publicly available due to the relatively small size of the elite leadership community within Swedish athletics, which increases the risk of participant identification. Pseudonymised data, where the preservation of anonymity can be assured, is available from the corresponding author on reasonable request.
