# Supplementary material for: Understanding safe sport as a public health practice: a strong structuration theory analysis of Swedish elite athletics sport personnel
Source: BMC Public Health. 2026 Sep 30;26:2949. doi: 10.1186/s12889-026-29709-8 (PMC13660012; doi:10.1186/s12889-026-29709-8)
Supplement: Supplementary file 1 [file 12889_2026_29709_MOESM1_ESM.pdf]

Supplementary File 1

SEMI-STRUCTURED INTERVIEW GUIDE: ATHLETICS ELITE SPORT PERSONNEL

(translated from Swedish to English)

Background questions:

- Age
- Gender
- Sport
- What percentage of your time do you spend working as a coach/leader within the current development environment? Do you have any other coaching or leadership roles?
- Could you tell me a little about your background as a coach, leader, or sport practitioner
- What experience do you have of coaching elite athletes at your current level of practice (junior/senior)?
- Could you briefly describe your coaching and/or leadership philosophy?

Part 1 (conceptualisation and understanding of safe sport)

- What comes to mind when you hear the term "safe sporting environments"?
- How would you describe the current sporting environment in which you operate in terms of how safe you perceive it for the athletes?
- Are there aspects of the current sporting environment that you believe contribute to making it safe for the athletes?
- Are there aspects of the current sporting environment that you believe contribute to making it unsafe for athletes?

Part 2 (development of safe and healthy sport systems)

- How would you describe an optimal sport environment that feels safe and healthy?

  Example probes:
  - Leaders/Coaches, Training groups, Communication, Support from significant others and the wider environment, Performance climate, Coordination between key stakeholders (e.g., school, family, coaches, and national team staff), Areas of development fostered within the environment

- Do you perceive that the sport environment in which you operate influences the mental health and wellbeing of those involved, either positively or negatively? Please provide examples.
- What do you believe is important within an elite sport environment for an athlete to maintain or improve their mental health over time? What do you believe are the

key risk factors within the sport environment that may contribute to mental health problems among athletes?

- Do you have any suggestions for how the sport environment in which you operate could be further developed to ensure that it is both safe and supportive for athletes while also providing optimal conditions for their development and performance?
